# Permanent Cardiac Pacing in the Fontan Population: A Contemporary Review of Indications, Approaches, and Outcomes

**DOI:** 10.1016/j.jacadv.2025.101667

**Published:** 2025-03-20

**Authors:** Bret L. Pinsker, Jeremy P. Moore, Thomas M. Bashore, Richard A. Krasuski

**Affiliations:** a Department of Medicine, Duke University Medical Center, Durham, North Carolina, USA; b Ahmanson/UCLA Adult Congenital Heart Disease Program, UCLA Health System, Los Angeles, California, USA; c Department of Cardiovascular Medicine, Duke University Medical Center, Durham, North Carolina, USA

**Keywords:** bradyarrhythmia, congenital, Fontan, pacing

## Abstract

Following the Fontan operation, electrophysiologic abnormalities requiring pacemaker implantation are common, consisting of sinus node dysfunction, complete atrioventricular block, and electromechanical dyssynchrony. Pacemaker implantation in this population can be challenging, as transvenous access to the cardiac chambers is often limited and may increase the risk of thromboembolism. Consequently, epicardial lead placement continues to be the default approach at most centers. Furthermore, permanent cardiac pacing has been associated with poor outcomes in this population (including an increased need for cardiac transplantation and death), even though it may be, depending on the approach, of great benefit for many individuals. Fortunately, improved understanding of the differential effects of cardiac pacing and novel approaches related to implantation have been developed and have increased their application to a growing number of patients. This review highlights the indications for pacing, methods to facilitate lead implantation, and associated outcomes in Fontan patients requiring permanent cardiac pacing.

## Highlights

• Pacemaker implantation following the Fontan procedure is common.
• Our understanding of the effects of cardiac pacing in this population is growing.
• Advances in lead implantation have expanded the eligibility of pacing in various Fontan subtypes.

### The Fontan procedure

**Figure 1:**
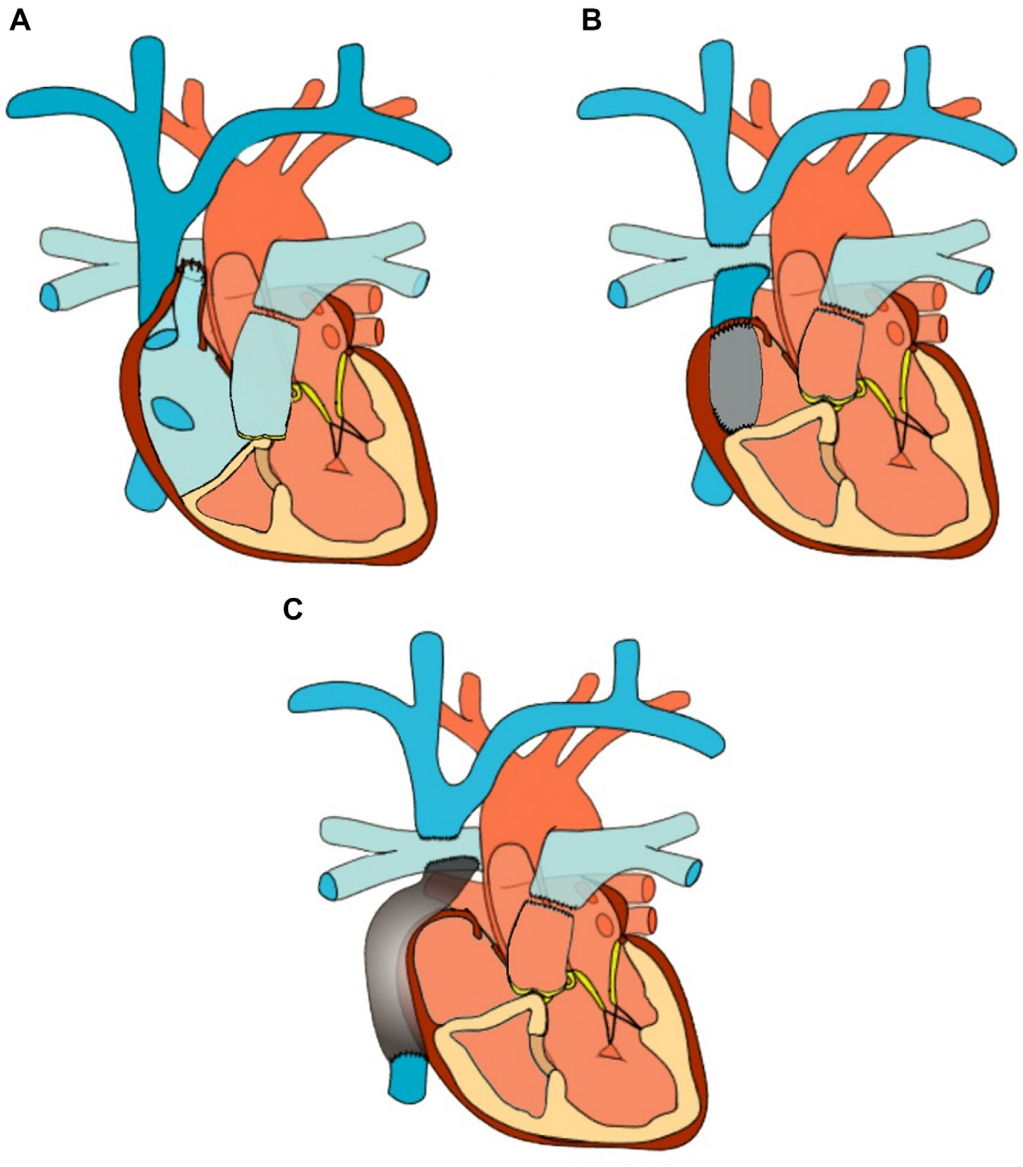
Common Types of Fontan Operative Procedures (A) A classic, atriopulmonary Fontan where the right atrial appendage is routed to the right pulmonary artery. (B) A lateral tunnel Fontan where the inferior vena cava is routed to the right pulmonary artery through the right atrium. The Glenn procedure routes the superior vena cava to the right pulmonary artery. (C) An extracardiac Fontan where the inferior vena cava is routed over the right atrium to the right pulmonary artery.

The Fontan procedure is the culmination of a series of operations intended to palliate various forms of single-ventricle physiology. For most patients, initial operations involve maneuvers to ensure a reliable degree of pulmonary and/or systemic blood flow. The second surgical correction, usually the “bidirectional Glenn” operation, begins the process of separating systemic and pulmonary venous blood flow by directly connecting the superior vena cava and the pulmonary artery (PA). The final stage of surgical correction is the Fontan operation, where the process is completed by routing inferior vena cava blood flow to the PA. Early iterations (“classic” or atriopulmonary (AP) Fontan) accomplished this by directly anastomosing the native right atrium (RA) to the PA. This approach was later abandoned due to subsequent maladaptive hemodynamics that promotes right atrial dilation, thrombus formation, and atrial arrhythmia.^1^ Consequently, modern total cavopulmonary connection approaches typically incorporate either a “lateral tunnel” (LT) that baffles inferior vena cava flow through the morphologic RA to the PA or employs an “extracardiac” (EC) conduit, whereby the RA is completely bypassed. The various approaches to the Fontan procedure are highlighted in Figure 1.

### Bradyarrhythmia in Fontan physiology

Arrhythmia is common among patients with Fontan physiology, with some cohorts (predominantly patients after the AP Fontan) estimating the cumulative 30-year incidence for arrhythmia or arrhythmic intervention at around 45%.^2^ Bradyarrhythmia, including sinus node dysfunction (SND), junctional rhythm, and less commonly atrioventricular (AV) block, accounts for a significant portion of this arrhythmia burden.^3^^,^^4^ The gradual development of bradyarrhythmia in this population is poorly understood, but is probably a consequence of overlapping mechanisms. In some cases, congenital malformations of the sinus node or AV conduction system may contribute, whereas in others, the Fontan procedure itself may facilitate bradyarrhythmia. After the AP Fontan for instance, massive morphologic RA dilatation and fibrosis may injure the sinus node complex. On the other hand, mechanisms for LT and EC Fontan patients include myocardial stretch and fibrosis and surgical manipulation near the cavoatrial junction with resultant injury to the sinus node artery or its efferent autonomic inputs.^5^, ^6^, ^7^ Lastly, AV block can develop when ventricular septal defect enlargement is attempted at early surgical repair or as a sequela of congenitally tenuous cardiac conduction system anatomy.^6^

In cases of clinically significant bradyarrhythmia, patients are generally referred for a permanent pacemaker (PPM). In fact, PPM placement is the most common reason for surgical reintervention in patients after the Fontan operation, with some reports estimating that around 15% of patients after total cavopulmonary connection will receive a PPM within 10 to 15 years.^8^^,^^9^ Decisions regarding the approach to implantation are often complex, as the lack of simple pathways from the central veins to atrial and ventricular myocardium limits standard transvenous options and prompts consideration of a surgical approach. Furthermore, our understanding of permanent cardiac pacing in this population remains quite limited. This review focuses on the rhythm abnormalities that require PPM placement in the Fontan population, the corresponding implant strategies, and long-term outcomes (Central Illustration).

**Central Illustration:**
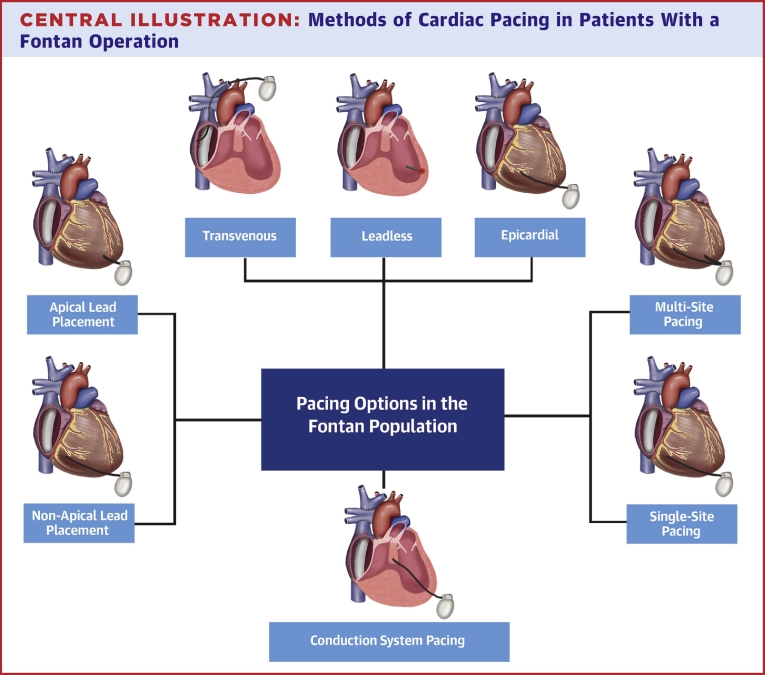
Methods of Cardiac Pacing in Patients With a Fontan Operation

## Atrial pacing: indications, lead implantation strategies, and outcomes

### Sinus node dysfunction

SND is a generic term that encompasses a variety of related phenomena, including sinus bradycardia, junctional rhythm, sinus pauses, and chronotropic incompetence. Among patients after total cavopulmonary connection, the long-term prevalence of SND ranges from 21% to 58%.^10^^,^^11^ Studies have shown that SND may lead to elevated pulmonary venous pressure, elevated Fontan circuit pressure, and reduced cardiac output.^12^ Over time, these changes may promote failure of Fontan physiology with concomitant Fontan-associated liver disease. Similarly, in cases where junctional rhythm replaces sinus rhythm, cardiac output is reduced, and retrograde atrial contraction during ventricular systole can be transmitted to the Fontan circuit.^13^

Whether or not the approach to Fontan palliation influences the risk of SND remains unclear. Formal comparisons of the LT and EC approaches have generally been limited by short durations of follow-up and variability in surgical strategies.^14^, ^15^, ^16^, ^17^ Consequently, these studies have demonstrated conflicting results, and no conclusions can currently be made regarding the superiority of either approach with regard to the development of SND.

### Indications for atrial pacing

Atrial pacing is generally accepted to be beneficial for symptomatic Fontan patients with SND. The most recent 2018 American College of Cardiology/American Heart Association/Heart Rhythm Society Guideline on the Evaluation and Management of Patients With Bradycardia and Cardiac Conduction Delay include atrial pacing as a Class I recommendation in this context.^18^ While such recommendations are helpful, it may be exceedingly difficult to determine when Fontan patients are truly “symptomatic.” Many are accustomed to living with severe, functional limitations for the entirety of their lives, and such individuals may experience significant clinical improvement with pacing. Conversely, nonspecific symptoms attributed to mild bradycardia may not improve.

In cases of asymptomatic SND, indications for PPM implantation are less certain. Given inherent risks associated with PPM placement after the Fontan operation, providers are often hesitant to refer such patients for a PPM. The decision to undergo an invasive procedure must be weighed against the poorly understood effects of chronic bradycardia and junctional rhythm on Fontan physiology. Currently, the 2014 Pediatric and Congenital Electrophysiology Society (PACES)/HRS Expert Consensus Statement on Arrhythmias in Adults with Congenital Heart Disease provides a class IIa recommendation for permanent pacing in patients with a resting heart rate <40 beats per second, ventricular pauses >3 seconds, impaired hemodynamics resulting from the bradyarrhythmia, and/or for the prevention of intra-atrial reentry tachycardia.^6^ Although similar recommendations (Class IIa) were included in the 2021 PACES expert consensus statement, there is a paucity of data that support such thresholds.^19^ Additional work is needed to better understand optimal metrics for PPM implantation; and while current guidelines may help providers identify patients with the most to gain from pacing, implantation must always be considered on a case-by-case basis.

Despite established physiologic importance, management of asymptomatic junctional rhythm after the Fontan operation is perhaps most controversial. In a survey of 154 electrophysiologists and congenital cardiologists, most felt that junctional rhythm in an asymptomatic Fontan patient was a benign finding, with consideration of further evaluation only after the development of symptoms. However, respondents also stated that they would place a PPM in a Fontan patient with junctional rhythm undergoing cardiac surgery for unrelated reasons, even when asymptomatic.^20^

### Lead implantation

The epicardial approach represents the conventional pacing strategy among Fontan patients with SND. Unfortunately, this requires either repeat sternotomy or thoracotomy. In addition, chronic epicardial lead performance is often unsatisfactory, with higher pacing thresholds, lower sensing amplitudes, and more rapid battery depletion.^21^ Lead failure is particularly common, with some studies estimating that nearly 40% of leads fail within 5 years of implantation.^22^ Consequently, multiple repeat operations may be required, increasing the associated morbidity of this approach.

**Figure 2:**
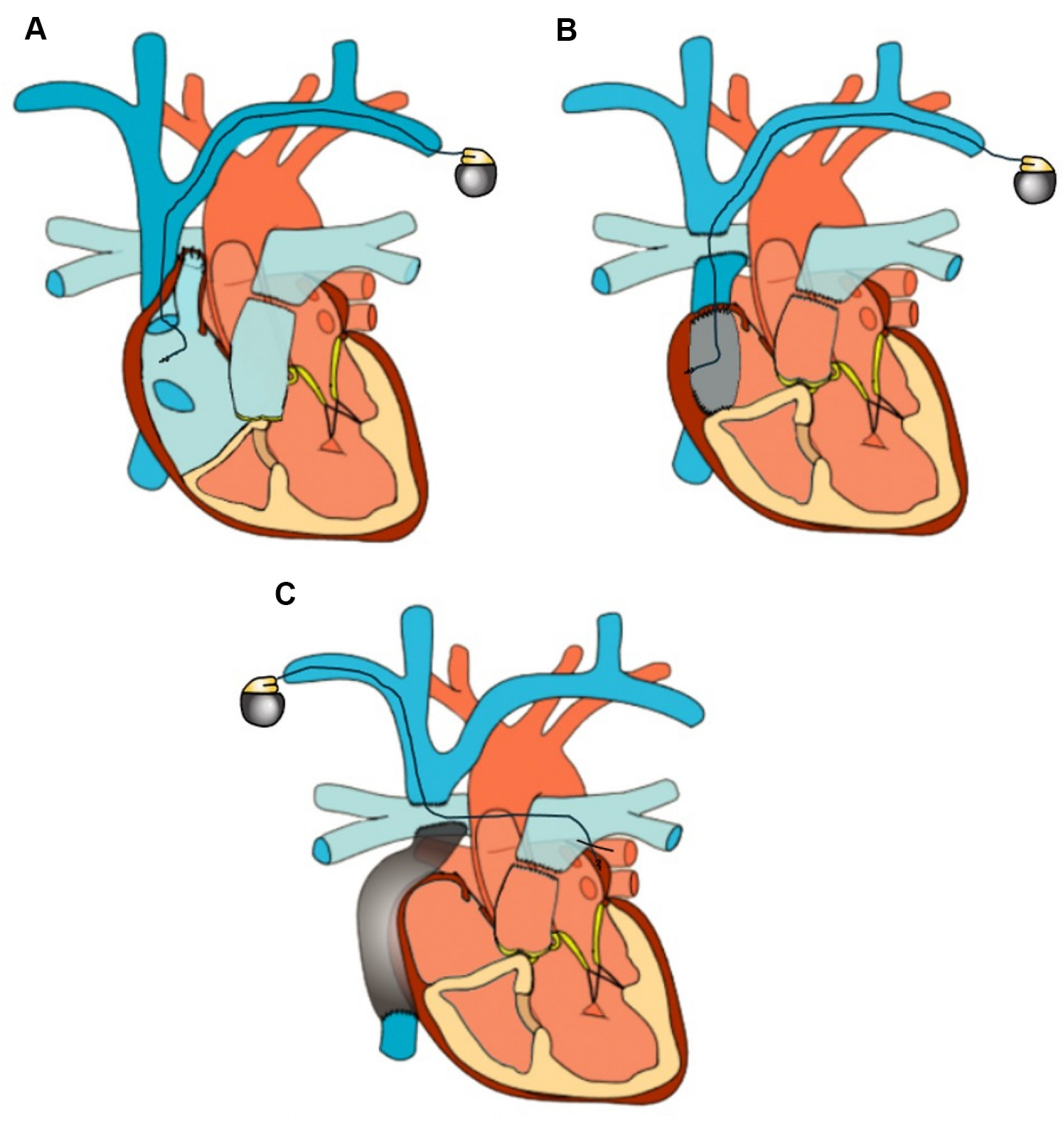
Examples of Nonepicardial (Transvenous) Atrial Pacing Options (A) A transvenous lead in the atrial mass in a patient with an atriopulmonary Fontan. (B) A transvenous lead pacing the morphologic, right atrial tissue forming the lateral tunnel. (C) A “transpulmonary” approach to atrial pacing is displayed. A puncture through the inferior aspect of the left pulmonary artery allows access to the left atrial epimyocardium. Prior iterations of this pacing technique have involved more distal lead placement situated in the pulmonary venous atrium.

Accordingly, less invasive approaches to establish atrial pacing have been proposed and are commonly utilized. Transvenous lead placement, for example, mitigates many of the aforementioned risks associated with epicardial pacing, and is associated with superior pacing characteristics as compared to epicardial leads.^21^ Furthermore, after both the AP and LT (but not EC) Fontan operations, direct transvenous atrial lead placement is usually possible (Figure 2). Recently, the “transpulmonary approach” modification for atrial lead placement after the EC Fontan has expanded eligibility for transvenous pacing. This involves a puncture through the inferior aspect of the PA to enter the adjacent left atrial chamber, or lead fixation at the atrial epimyocardium (Figure 2).^23^, ^24^, ^25^ Although these strategies offer less invasive methods for atrial pacing, they are currently only performed at highly specialized centers.

Not surprisingly, transvenous lead placement is associated with its own unique limitations. Fontan pathway obstruction may develop owing to lead placement within the surgically reconstructed venous pathway, and thrombus may form in relation to the intravascular leads. Among a cohort of 232 Fontan patients with various pacemaker approaches, those with transvenous leads displayed the highest burden of thrombus and associated embolic events (stroke, transient ischemic attack, and pulmonary embolus).^26^ However, this difference was not statistically significant, and the risk of thromboembolism was greatly reduced with anticoagulation. While these results highlight relative safety of transvenous leads with concurrent anticoagulation, patient adherence and clinically significant bleeding with long-term therapy remain important considerations. Finally, leadless approaches to atrial pacing after variants of the Fontan operation have recently been described and may be increasingly utilized as experience with this newer technology grows.^27^^,^^28^

### Outcomes associated with atrial pacing

Limited reports examining the acute hemodynamic impact of atrial pacing in Fontan patients are favorable (Table 1). In particular, atrial pacing in the acute, postoperative setting is associated with significantly improved cardiac indices and lower atrial pressure.^29^ The physiologic benefits of atrial pacing in this population have also been confirmed in studies involving baseline junctional rhythm, where echocardiographic measures before and during a period of atrial pacing demonstrate higher cardiac indices, improved ventricular filling patterns, and less pulmonary venous flow reversal.^30^ Finally, assessment of patients with junctional rhythm undergoing cardiac catheterization demonstrated decreased left atrial pressure, increased cardiac index, and increased pulmonary blood flow with atrial pacing.^31^

**Table 1:** Studies Assessing the Impact of Pacing on Outcomes in the Fontan Population

	First Author	Design	Sample Size	Findings in Fontan Population
Atrial pacing	Barber et al.	Single-center crossover	21	Atrial pacing was associated with improved cardiac output in the postoperative period following Fontan completion
	Yang et al.	Single-center prospective cohort	9	Atrial pacing resulted in higher cardiac indices, lower E/e' ratios, and a decreased likelihood of pulmonary venous flow reversal in patients with an underlying junctional rhythm
Ventricular pacing	Barber et al.	Single-center crossover	21	Asynchronous ventricular pacing was associated with maladaptive hemodynamic parameters in the postoperative period following Fontan completion
	Bulic et al.	Multicenter retrospective cohort	22	Ventricular pacing conferred an increased risk of ventricular dysfunction, AV valvular regurgitation, and cardiac transplantation
	Chubb et al.	Multicenter retrospective cohort	213	An increased burden of ventricular pacing in patients with single ventricle physiology was associated with an increased risk of cardiac transplantation and death
	Fishberger et al.	Single-center retrospective cohort	46	Pacing offers no survival benefit, but dual-chamber pacing is associated with decreased mortality when compared to asynchronous ventricular pacing
	Kodama et al.	Single-center retrospective cohort	27	A higher proportion of ventricular pacing was associated with increased serum BNP levels and an increased risk of cardiac transplantation and Fontan failure
Multisite pacing	Chubb et al.	Multicenter retrospective cohort	20	CRT did not result in a decreased risk of cardiac transplantation or death
	O'Leary et al.	Single-center retrospective cohort	62	Compared to patients with single-site ventricular pacing, CRT was not associated with a mortality benefit or decreased likelihood of cardiac transplantation
	Joyce et al.	Single-center retrospective cohort	27	Indices of systolic and diastolic cardiac function improved in patients with single-ventricle physiology that received multisite pacing

AV = atrioventricular; BNP = brain natriuretic peptide; CRT = cardiac resynchronization therapy.

Unfortunately, aside from isolated case reports, data examining long-term clinical outcomes of atrial pacing after the Fontan operation are limited. In a patient with protein losing enteropathy refractory to other treatment modalities, 3 weeks of atrial pacing resulted in increased cardiac output as well as sustained protein losing enteropathy resolution up to 6 months.^32^ Although such anecdotal evidence points toward benefit with atrial pacing, greater study is needed to assess the reproducibility of these results.

## Ventricular pacing: indications, modifiable risk factors, and outcomes

### Atrioventricular block

AV block after the Fontan operation is estimated to affect between 2 and 16% of patients.^33^ Risk is related to both the iatrogenic sequelae of prior cardiac operations, as well as propensity for AV conduction abnormalities in the setting of specific forms of congenital anatomy.^34^ The latter is especially common for patients with left atrial isomerism, where absence of a connecting AV bundle may exist at birth; and for those with l-looped ventricles (such as the classic form of double inlet left ventricle), where a tenuous nonbranching bundle is susceptible to progressive fibrosis and AV block over time.^6^ Among patients with Fontan physiology, AV block can result in heart failure symptoms, arrhythmic syncope, and sudden cardiac death.

### Indications for ventricular pacing

Although the prevalence of high-grade or complete AV block in the Fontan population is less than that of SND, some studies suggest it to be the more common indication for PPM implantation.^35^^,^^36^ This may be driven by the greater risks associated with AV block, even when asymptomatic. Accordingly, the 2018 American College of Cardiology/American Heart Association/Heart Rhythm Society Guidelines provide Class I recommendations for PPM placement in adults with congenital heart disease who demonstrate symptomatic AV block, as well as AV block with wide QRS escape rhythm, sustained heart rates <50 beats/min, complex ventricular ectopy, or ventricular dysfunction, regardless of symptoms.^18^ The 2021 Pediatric Guidelines provide similar recommendations for patients with symptomatic, high-degree AV block, as well as asymptomatic, complete AV block when the average heart rate is <60 to 70 beats/min.^19^

### Outcomes associated with single-site ventricular pacing

Regardless of the status of AV conduction, chronic, single-site ventricular pacing in the Fontan population has been associated with poor outcomes. Originally, it was demonstrated that, at best, single-site ventricular pacing in patients with Fontan physiology offers no survival benefit.^36^ More recently, large observational studies have demonstrated increased AV valvular regurgitation, worsening ventricular dysfunction, and worse functional status, as well as increased rates of cardiac transplantation and death.^29^^,^^37^, ^38^, ^39^ Although adverse outcomes are more common in patients receiving any type of ventricular pacing, they are most common among those with single-chamber devices, where AV dyssynchrony is most pronounced.^29^

Initial studies examining single-site ventricular pacing were unable to establish cause-and-effect with adverse outcomes. Although some found a high burden of ventricular pacing correlated with higher serum brain-natriuretic peptide levels, an increased risk of heart transplantation, and Fontan failure, small sample sizes, and single-center cohorts limited their generalizability.^40^ Furthermore, Fontan patients frequently suffer from a variety of comorbidities that negatively impact their prognosis, irrespective of the need for ventricular pacing. Whether PPM implantation itself contributed to these outcomes or was simply a confounding variable among those destined for adverse outcomes was previously unresolved. Recently, Chubb et al^39^ published a multicenter observational study involving 236 Fontan patients with an implanted PPM. A clear “dose-dependent” relationship was demonstrated between single-site ventricular pacing and adverse outcomes. Specifically, increasing requirement for ventricular pacing was associated with incremental risk for heart transplantation and Fontan failure.^39^ Such data strongly implicate ventricular pacing itself with adverse clinical outcomes, likely via its effects on electromechanical dyssynchrony and adverse hemodynamics.

For Fontan patients with AV block, there is surprisingly limited data to support dual-chamber pacing. Beyond the intuitive superiority associated with this approach, some clinical studies suggest improved hemodynamics when dual-chamber pacing is implemented as compared to single-site ventricular pacing.^29^ Better clinical outcomes have also been reported for patients with dual-chamber pacing as compared to those with single-site ventricular pacing.^36^ Such findings are not surprising, given the fragile physiology of the Fontan circulation and the importance of AV synchrony in promoting optimal cardiac output. However, whether this perceived benefit warrants dual-chamber pacing for all patients with Fontan physiology must be weighed in relation to the established surgical morbidity associated with atrial lead placement. A summary of the studies that have examined the impact of ventricular pacing in patients with Fontan physiology is displayed in Table 1.

### Lead placement

**Figure 3:**
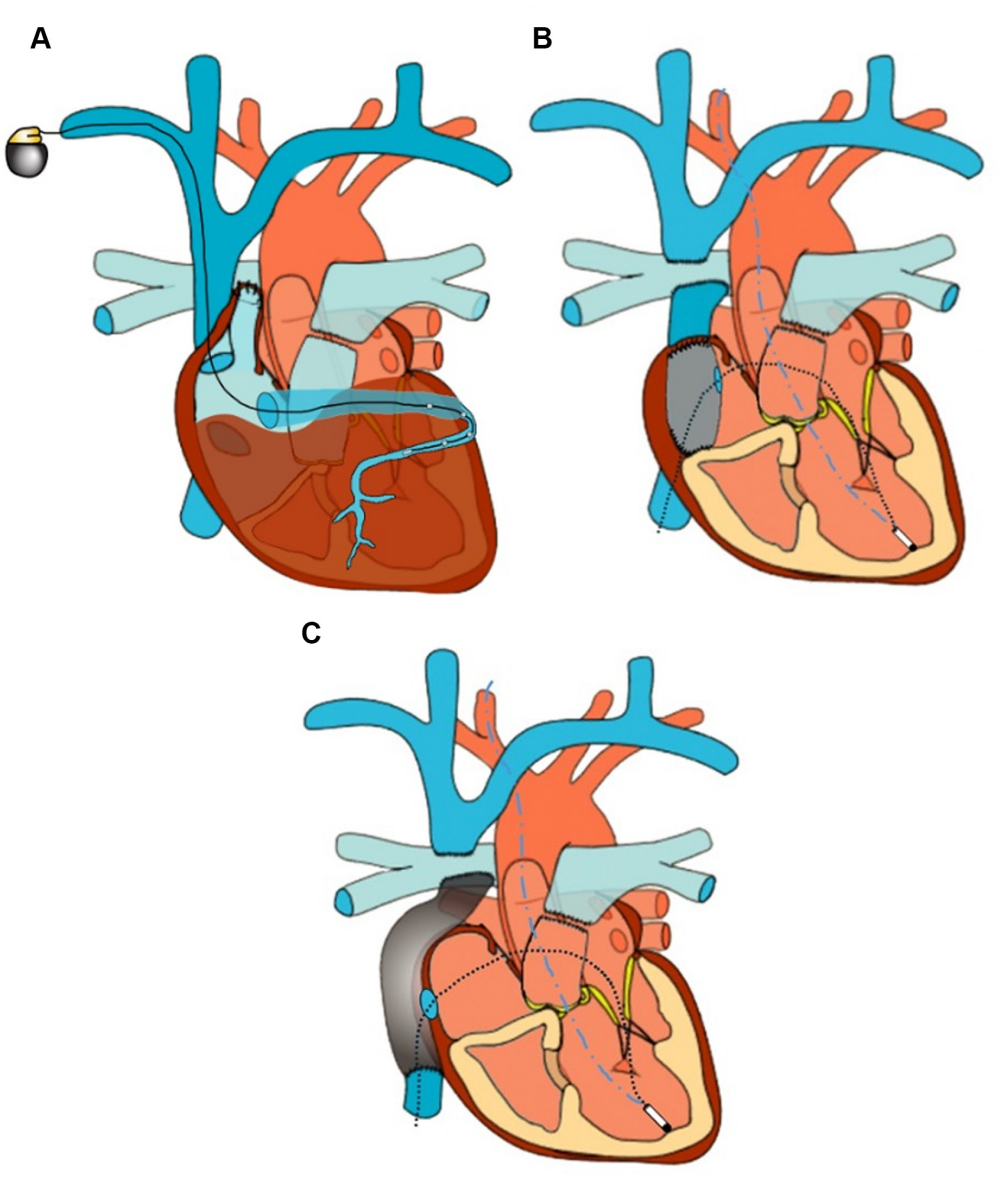
Potential Options for Ventricular Pacing (A) A transvenous lead coursing through the coronary sinus in a patient with an atriopulmonary Fontan. (B and C) Transcarotid approach to leadless pacemaker implantation (dashed blue lines). Fenestration of the Fontan pathway allows for an alternative method of access to the systemic ventricle (dashed black lines).

Like atrial pacing, ventricular lead placement has traditionally been achieved via an epicardial approach. In specific situations, such as after the AP Fontan, transvenous lead placement in a large, but tortuous coronary sinus may be possible (Figure 3).^41^, ^42^, ^43^ Although transvenous ventricular lead placement through a trans-baffle puncture has also been described in a patient with a LT Fontan, this imposes a risk of thromboembolism.^44^ As such, endocardial lead placement in patients with congenital heart disease and intracardiac shunts is associated with a class III recommendation (harmful) and should only be considered in select cases.^19^

Although previously unavailable, the recent introduction of leadless cardiac pacing has expanded the cohort of patients that are eligible for endocardial pacing. In particular, reports involving EC Fontan patients have demonstrated the feasibility of transcarotid and transconduit approaches to leadless ventricular pacing (Figure 3).^45^^,^^46^ Although these approaches may confer substantial benefits, imperfect AV synchrony, electromechanical dyssynchrony, and residual thromboembolic risk remain of concern.

### Lead location

Significant effort has been aimed toward identifying and avoiding the maladaptive effects of ventricular pacing after the Fontan operation. In this context, lead location has received considerable attention. Several studies have now clearly demonstrated that nonapical lead placement is associated with increased serum levels of brain-natriuretic peptide, cardiac transplantation, and death.^39^^,^^40^ Unfortunately, apical lead placement is not easily achieved in many patients after multiple cardiac operations. Adequate locations for lead placement are often limited by scarring from prior operations with suboptimal pacing thresholds in large regions of epimyocardium.

### Multisite pacing

**Figure 4:**
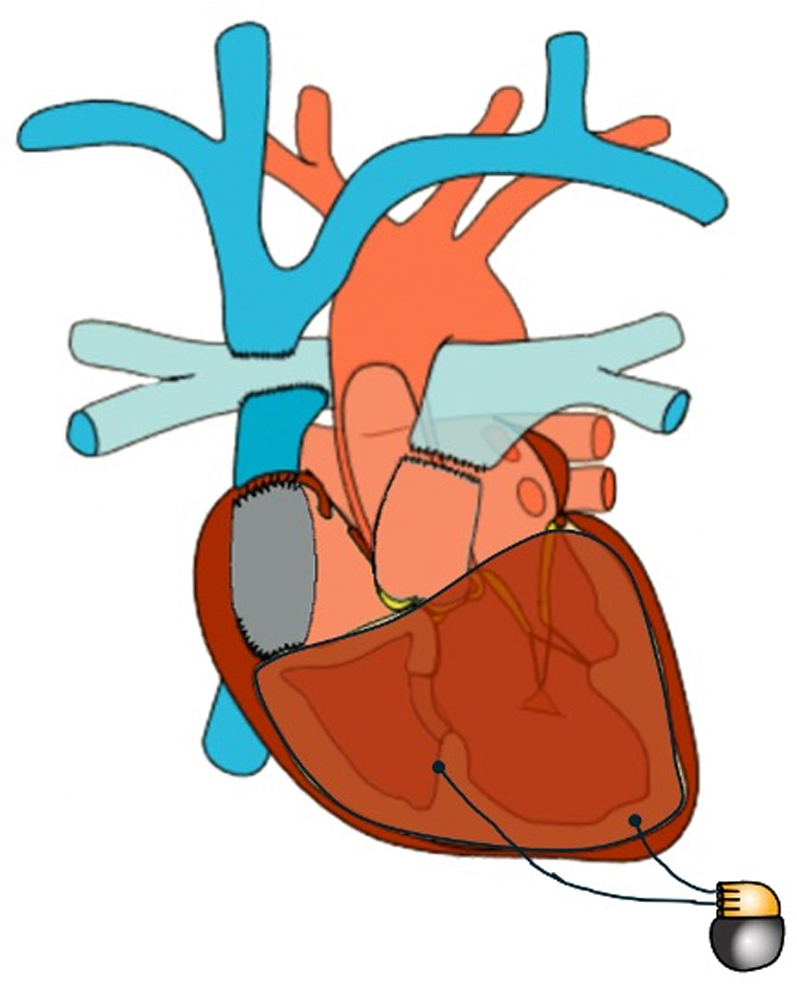
Example of Multisite Ventricular Pacing Multisite ventricular pacing is displayed with 2 epicardial leads.

Cardiac resynchronization therapy (CRT), also known as “multisite pacing,” among Fontan patients, aims to restore ventricular synchrony by simultaneous (or near simultaneous) electrical excitation at disparate ventricular locations. An example of multisite pacing is displayed in Figure 4. Although preliminary findings are encouraging, no study dedicated to Fontan physiology has yet demonstrated superiority of multisite pacing over other strategies (Table 1). In the previously mentioned analysis by Chubb et al,^39^ 20 patients with multisite pacing were compared to 216 patients with single-site pacing. Although no significant difference in heart transplant-free survival was observed between groups, the vast majority of multisite pacing patients had undergone delayed “upgrade” from single-site pacing.^39^ Similarly, a subgroup analysis of 19 patients with single ventricular physiology by the same authors demonstrated similar results, again without statistical significance.^47^ Finally, O'Leary et al^48^ demonstrated borderline (*P* = 0.08) improvement in transplant-free survival in a single-center cohort of Fontan patients undergoing multisite pacing. It is likely that all these clinical studies o f CRT in Fontan patients have been underpowered to detect differences in these important clinical outcomes. Supporting this possibility, Joyce et al^49^ recently assessed advanced, quantitative metrics of ventricular function in a cohort of 19 patients following the EC Fontan operation. When compared to conventional dual-chamber pacing, systolic and diastolic ventricular function were significantly improved up to 5 years with multisite pacing.^49^

### Conduction system pacing

Conduction system pacing refers to pacing methods that target the His bundle or left bundle branch areas, with a goal of improving ventricular synchrony. Such physiologic pacing has been demonstrated to be at least equivalent to biventricular CRT among patients with congenital heart disease, but has not been previously considered to be feasible after the Fontan operation.^50^ Interestingly, successful conduction system pacing was recently achieved via subxiphoid, transpericardial approach in a patient with a LT Fontan and was associated with significant electrocardiographic response.^51^ Inadvertent conduction system capture was also likely present in at least one other prior Fontan report.^46^ Although promotion of ventricular synchrony via conduction system pacing may offer significant benefit to the Fontan population, additional work is necessary to better understand its value.

## Conclusions

The effects of pacing in the Fontan population are increasingly understood. Although atrial pacing is acutely of established hemodynamic benefit, clinical thresholds for pacing referral among patients with asymptomatic SND or junctional rhythm remain unclear. On the other hand, while permanent ventricular pacing is detrimental to the fragile Fontan physiology, these effects are potentially mitigated by either apical lead placement or multisite pacing. Finally, advances in procedural techniques and novel technologies have challenged the notion that epicardial lead placement should be the default option for all patients requiring permanent cardiac pacing after the Fontan operation.

## Funding support and author disclosures

Dr Krasuski has served as a consultant for Actelion/Janssen Pharmaceuticals, Bayer, Gore Medical, Medtronic, and Neptune Medical; has received research funding from the Adult Congenital Heart Association and Actelion/Janssen Pharmaceuticals; and has served as a principal investigator for trials with Edwards Lifesciences, Gradient Denervation Technologies, and Medtronic. All other authors have reported that they have no relationships relevant to the contents of this paper to disclose.
